# Diamond Grinding Wheel Condition Monitoring Based on Acoustic Emission Signals

**DOI:** 10.3390/s21041054

**Published:** 2021-02-04

**Authors:** Guo Bi, Shan Liu, Shibo Su, Zhongxue Wang

**Affiliations:** College of Aerospace Engineering, Xiamen University, Xiamen 361005, China; liushan@stu.xmu.edu.cn (S.L.); 19920181152233@stu.xmu.edu.cn (S.S.); 19920201151467@stu.xmu.edu.cn (Z.W.)

**Keywords:** acoustic emission, condition monitoring, grinding process, diamond wheel, brittle materials

## Abstract

Acoustic emission (AE) phenomenon has a direct relationship with the interaction of tool and material which makes AE the most sensitive one among various process variables. However, its prominent sensitivity also means the characteristics of random and board band. Feature representation is a difficult problem for AE-based monitoring and determines the accuracy of monitoring system. It is knottier for the situation of using diamond wheel grinding optical components, not only because of the complexity of grinding process but also the high requirement on surface and subsurface quality. This paper is dedicated to AE-based condition monitoring of diamond wheel during grinding brittle materials and feature representation is paid more attention. AE signal of brittle-regime grinding is modeled as a superposition of a series of burst-type AE events. Theory analysis manifested that original time waveform and frequency spectrum are all suitable for feature representation. Considering the convolution form of b-AE in time domain, a convolutional neural network with original time waveform of AE signals as the input is built for multi-class classification of wheel state. Detailed state division in a wheel’s whole life cycle is realized and the accuracy is over 90%. Different from the overlapping in time domain, AE components of different crack mechanisms are probably separated in frequency domain. From this point of view, AE spectrums are more suitable for feature extraction than the original time waveform. In addition, the time sequence of AE samples is essential for the evaluation of wheel’s life elapse and making use of sequential information is just the idea behind recurrent neural network (RNN). Therefore, long short-term memory (LSTM), a special kind of RNN, is used to build a regression prediction model of wheel state with AE spectrums as the model input and satisfactory prediction accuracy is acquired on the test set.

## 1. Introduction

Grinding wheel wear estimation is essential to ensure quality control of the ground surface and reduce downtime and tool costs. There are two types of tool condition estimation methods, i.e., directly and indirectly. Directly detecting wheel topography is reliable and objective, but limits by environmental conditions and cannot be applied in real-time monitoring system. On the other hand, indirect methods by measuring physical quantities during machining process are real time and convenient. Wheel condition monitoring techniques are attracting increasing amounts of research attention and monitoring systems have been a necessary segment of modern machining equipment [1,2,3,4]. Various physical quantities have been used for tool condition monitoring. The most common-used variables on wheel condition monitoring include vibration, force, temperature and acoustic emission (AE). AE is an elastic stress wave generated within a material when it undergoes deformation and fracture [5]. Different from other variables which are synthetic representation of machining process, AE signals can be released from any tiny interaction behaviors between tool and material [6]. The range of frequency is much higher than the environmental frequencies. The sensitivity and immunity from interference make AE signals quite suitable for tool condition monitoring. AE signals are used to evaluate grinding wheel conditions in many studies [7,8,9].

AE signals dramatically change for the occurrence of a sudden damage on the tool during machining process. Thresholding methods have been successfully employed to monitor breakage or wear of tools with deterministic cutting edges [10,11]. However, for grinding process, the characteristics of wheel wear are not as typical as milling or drilling. AE signals are entirely random because abrasive grains protruding from wheel surface are random in shape, orientation and location. Various signal processing methods were employed for AE feature extraction. Sutowski [12] utilized an aloxite grinding wheel to grind tool steel; the experimental results showed the close relationship between the root-mean-square (RMS) value of AE and the cutting performance of grinding wheel. Stephenson [13] investigated ELID grinding of optical materials and used AE to detect wheel loading. AE energy will increase dramatically when wheel loading occurs. Aguiar [14] attempted to detect grinding burn in grinding steel material by an aluminum oxide grinding wheel. Various statistical parameters of AE were extracted for monitoring. Their studies showed that constant false alarm rate and ration of power were more sensitive than RMS ratio. Mokbel [15] picked up AE signals during grinding mild steel specimens by diamond grinding wheels with different bond types and grain sizes. Fourier transform was applied to acquire AE spectrum. The research showed that AE spectral amplitude was obvious increase for a dull wheel. Wheel dressing conditions were also considered in their experiments. The comparison analysis verified that dressing conditions had a prominent influence on AE features. Liao [16] collected AE signals in creep feed grinding alumina specimens with a resinoid-bonded diamond wheel. Discrete wavelet decomposition was employed for AE feature extraction. Satisfactory classification accuracy can be acquired based on the extracted features for high material removal rate condition. Yang [17] focused on grinding burn in up-grinding steel with vitrified aluminum oxide wheel. Additionally, Hilbert-Huang transform was used to decompose grinding AE signals into a series of intrinsic mode functions (IMFs). The first five IMFs were used to extract features for grinding burn detection. In fact, although many artificial features are effective for wheel state monitoring, feature extraction by using try and error is not adequate for the high accuracy monitoring. In order to select the appropriate features, Liao [18] investigated feature extraction and feature selection. AE signals were collected during grinding ceramic materials with a resin bonded diamond wheel. AE features were extracted both by discrete wavelet decomposition and autoregressive modeling. The three feature selection methods include the sequential forward floating selection, and two ant colony optimization-based feature selection methods based on different search strategies. Experimental results showed that lower classification error was acquired by using the selected features.

Due to the complexity of grinding process and AE mechanism, data-driven modelling is the predominant tendency for AE-based monitoring of wheel condition [19]. Lezanski [20] applied a neural network to select 8 typical features from a feature set which was composed of grinding operation parameters, vibration and AE features. The selected features were put into fuzzy logic-based systems for grinding wheel state classification during external cylindrical grinding process. Yang [21] constructed a binary classification system of wheel condition by support vector machine (SVM) algorithm. The input of the system was AE features extracted by discrete wavelet decomposition. Satisfactory classification accuracy was acquired for different cutting depth. Nakai [22] employed four types of neural networks for diamond wheel wear estimation during the grinding of advance ceramics. They were multilayer perceptron neural networks, radial basis function neural networks, generalized regression neural networks and adaptive neuro-fuzzy inference system. Features derived from AE and cutting power signals were taken as their inputs. The various types of networks complemented each other. Pandiyan [23] studies abrasive belt wear in robotized abrasive belt grinding process. Genetic Algorithm was used to select features derived from accelerometer, AE sensor and force sensor. Then, the selected features were put into a model based on SVM to judge the tool worn or not. Guo [24] introduced deep learning into grinding burn classification. A two-stage feature selection approach combining ReliefF and stacked sparse autoencoder network was proposed to select the best subset of features and establish an accurate classification model of grinding burn. Wavelet packet decomposition and ensemble empirical mode decomposition were applied to process the original force, acceleration, and AE signals. The experimental results suggested that the proposed approach outperforms other methods which were artificial neural network, SVM and sparse autoencoder network.

Almost all types of machine learning models have been employed for wheel condition monitoring. Various manual features extracted by conventional or advanced signal processing methods were attempted for good performance of models. Whether or not features are appropriate determines monitoring accuracy of grinding wheel condition. The majority of existing research attempts to select the most proper features from a feature set. To make sure to gain the optimal combination, there is a tendency of providing a huge amount of selected features. For instance, the reference [24] employed a total of 16,208 artificial features for feature selection. Feature engineering is a necessary preprocess for traditional machine learning models. Nevertheless, deep learning, as a prospective subfield of machine learning, can integrate feature extraction and selection in the model itself [25]. Recent advances in deep learning methods have achieved revolutionary success in tool condition monitoring [26]. In this paper, surface grinding brittle materials by diamond grinding wheel was focused and two types of deep learning models, convolutional neural network (CNN) and long-short-term memory (LSTM), were attempted to monitor the degradation of wheel condition. Based on the analysis of AE mechanism of grinding brittle material, original AE time waveform or AE frequency spectrum was taken as input of the models. Experiments were carried out through a whole life cycle of a diamond wheel and verified the performance of CNN and LSTM.

## 2. Characteristics of AE Signals during Grinding Brittle Materials

AE signals can be classified into two different types: continuous-type AE (c-AE) and burst-type AE (b-AE). C-AE is characterized by a long duration signal with variations in amplitude and frequency. B-AE is characterized by a short duration signal with high amplitude [27]. There are abundant AE sources during machining process. The formation of cracks and chips on materials, the removal and breakage of grains, and collision between them always induce b-AE. Elastic and plastic deformation, friction and flow noise is always associated with c-AE [28,29]. When using diamond wheel grinding brittle materials, the majority of materials are removed under brittle regime. Therefore, the predominant AE sources during the process are the initiation and prorogation of cracks. Lateral crack may extend to the surface of the ground material and cause material removal. Additionally, median crack may penetrate in the ground material and become subsurface damages [30]. Lateral crack and median crack are all associated with material properties, grain geometry and applied load [31,32].

The waveform of a b-AE is characterized by rapid damping of a super-high oscillation. Additionally, duration and frequency content depend on the motion of the crack tip and therefore, carry information about the mode of the crack [33,34,35]. It is reasonable to believe that several crack modes are predominant during grinding process because of the similar size of abrasive grains on wheel surface. A b-AE event can be simply simulated as $Ae^{-\alpha t}\cos\omega t$, where α determines attenuation speed and the sign ω is the oscillation frequency. AE signal of grinding brittle material can be represented as a superposition of a series b-AE events inspired on different time occasion. From the point view of statistics, some b-AE events may be induced by the same mode of crack and some of them probably share parameters. Therefore, AE signal can be represented as follows:

(1) $$x\left(t\right)=\sum_{m=1}^{M}\left\{\left[e^{-\alpha_{m}t}u\left(t\right)\cos\omega_{m}t\right]*\sum_{q=1}^{Q}A_{qm}\delta \left(t-t_{qm}\right)\right\}$$

where the sign t represents time and u(t) is the unit step signal. The sign δ is the delta function. The operational symbol ∗ is the convolution operation. The subscript *m* represents a certain kind of b-AE, and $\alpha_{m}$ and $\omega_{m}$ are the damping factor and oscillation frequency, respectively. The subscript *q* represents time occasions of b-AE events. $t_{qm}$ where *q* from 1 to Q, is the concrete time of the a specific kind of b-AE and $A_{qm}\geq 0$ is the corresponding amplitude.

As shown in Equation (1), there are two types of information in AE time waveform. The one is b-AE mechanism represented by the parameters $\alpha_{m}$ and $\omega_{m}$. The other is the occurrence time points. The degradation of wheel’s grinding performance results from the statistical change of grain state. The statistical change of grain state alters the proportion of various b-AE components in AE signals. In consideration of the convolution form of AE time waveform, convolution neural network (CNN) is tried to identify wheel state classes based on original AE time waveform in the next section.

Using convolution definition and the properties of delta function [36], the Equation (1) can be rewritten as follows:

(2) $$\begin{array}{cc} x\left(t\right) & =\sum_{m=1}^{M}\left\{\int_{-\infty }^{+\infty }e^{-\alpha_{m}\tau }u\left(\tau \right)\cos\omega_{m}\tau \sum_{q=1}^{Q}A_{qm}\delta \left(t-\tau +t_{qm}\right)d\tau \right\}\\ & =\sum_{m=1}^{M}\left\{\sum_{q=1}^{Q}A_{qm}\int_{\infty }^{+\infty }e^{-\alpha_{m}\tau }u\left(\tau \right)\cos\omega_{m}\tau \delta \left(t-\tau +t_{qm}\right)d\tau \right\} \end{array}\;=\sum_{m=1}^{M}\sum_{q=1}^{Q}A_{qi}e^{-\alpha_{m}\left(t-t_{qm}\right)}u\left(t-t_{qm}\right)\cos\omega_{m}\left(t-t_{qm}\right)$$

and the Fourier transform is:

(3) $$X_{half}\left(\omega \right)=\sum_{m=1}^{M}\frac{1}{\alpha_{m}+j\left(\omega -\omega_{m}\right)}\sum_{q=1}^{Q}\frac{A_{qm}}{2}e^{-j\omega t_{qm}}$$

where the subscript half means the positive half part of Fourier transform of x(t). For a specific component of b-AE with parameters $\alpha_{m}$ and $\omega_{m}$, the amplitude spectrum is $1/\sqrt{\alpha_{m}^{2}+{\left(\omega -\omega_{m}\right)}^{2}}$ which is naturally limited within a frequency span centered by $\omega_{m}$. AE components of a specific crack mode occurring on different time occasion are totally overlapped on the spectrum, whereas AE components of different crack mechanism are separated in frequency domain. Therefore, AE signals of grinding process exist several characteristic frequency bands with obvious energy distribution. The separation of different modes of crack provides more obvious information for wheel condition monitoring. Once the state of grains on wheel surface statistically changes, the transformation of the predominant crack mechanism probably changes the structure of AE frequency spectrum and its energy distribution.

## 3. Wheel’s Life Cycle Experiments

### 3.1. Experimental Instruments

**Figure 1:**
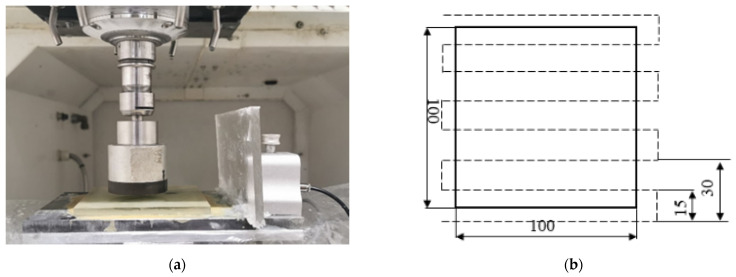
(**a**) experimental instruments; (**b**) grating scanning path.

**Figure 2:**
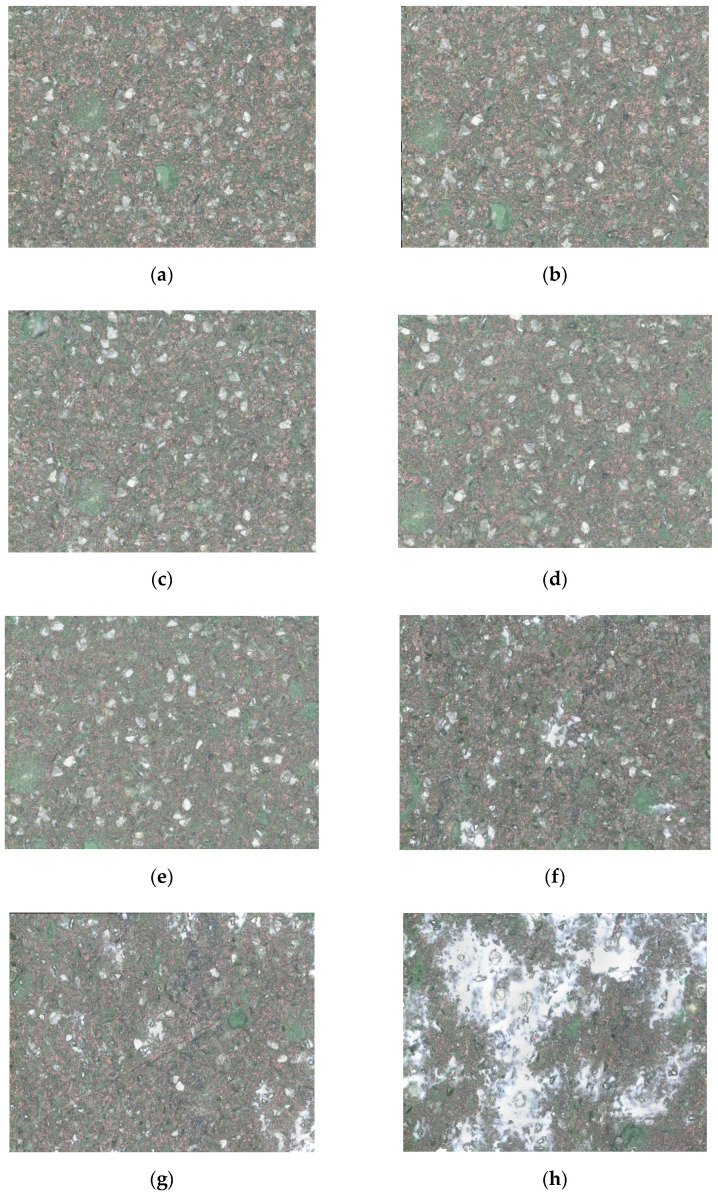
Wheel topography evolution during grinding. (**a**) the 1st node; (**b**) the 3rd node; (**c**) the 5th node; (**d**) the 9th node; (**e**) the 13th node; (**f**) the 15th node; (**g**) the 17th node; (**h**) the 19th node.

Surface grinding experiments were carried out on a M1.7 CNC machine of Shenyang Machine Tool Corporation of China as shown in Figure 1a. A customized diamond cup wheel was employed to grind Fused silica glasses HPFS 7980 (Coring, New York, NY, USA) The material is commonly used for large-scale optical lens. PE552 water-soluble cooling fluid (Shanghai YUANCH Optical Material Corporation, Shanghai, China) was used as grinding coolant. The workpiece size was 100 mm × 100 mm × 10 mm. Other basic parameters of the wheel and the workpiece are listed in Table 1. Grating scanning path was adopted during grinding, and the grinding path sketch is shown in Figure 1b. The wheel underwent a whole life cycle from just after dressing to its life end. Machining parameters and observation nodes are listed in Table 1. AE signals and wheel topography were acquired when material removal volume reached these nodes. The interval of the nodes was 0.1 cm^3^. The acquisition frequency of AE signal was 1 MHz and the time length of acquisition was about 10 s. The time interval of adjacent nodes was about 3 min. Time consumption of AE signal acquisition on each node was negligible compared with the interval. Therefore, it was reasonable to suppose unchangeable wheel state during AE acquisition. R50A AE sensor and PCI-2 AE acquisition system of Physical Acoustics Corporation were used to acquire AE signals. AE signal acquired on each node was divided into 10 ms length data segments which were labelled in the same class. The sequence order of all the 19 nodes were taken as data labels. Wheel topography images were also detected by VHX-5000 microscope (KEYENCE Corporation, IL, USA) on odd number nodes. Some of them are shown in Figure 2 and the magnification was 500 times. Wheel loading was predominant wear pattern companied with slight grain flattening for the used wheel. Sporadic blockages can be observed for the 13th node. Blockages were more obvious for the 15th node. Additionally, the situation got worse and worse for the following nodes. In the end, blockages existed in a continuous area on the wheel surface. During grinding optical materials, in order to avoid unexpected catastrophic damages on or under the ground surface, high stability of grinding performance of diamond wheel is necessary. Obvious changes on the wheel topography manifests that truing and dressing should be carried out.

### 3.2. Basic Analysis of AE Signals

**Figure 3:**
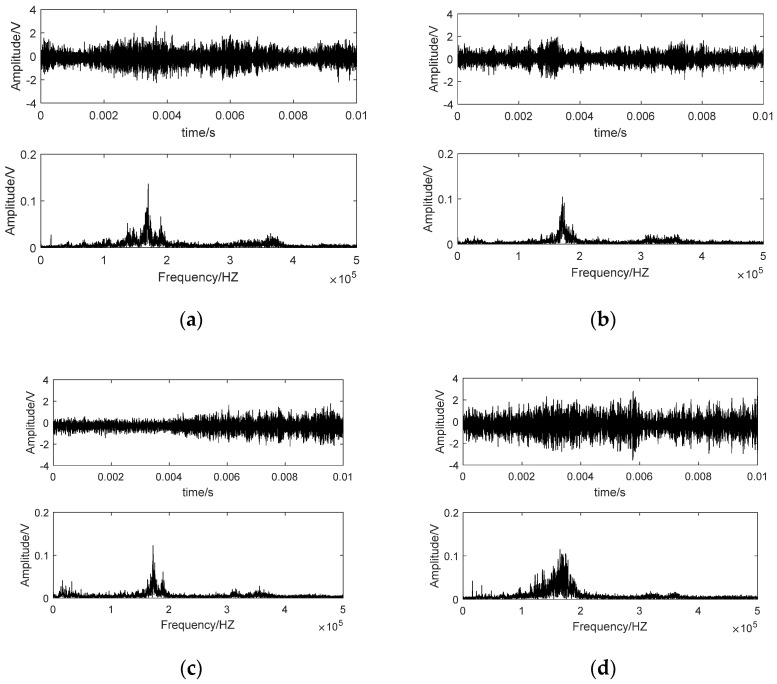

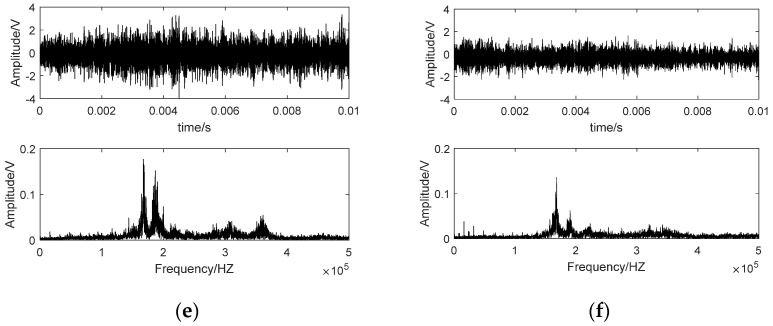
AE signals on different nodes. (**a**) the 1st node; (**b**) the 3rd node; (**c**) the 5th node; (**d**) the 13th node; (**e**) the 17th node; (**f**) the 19th node.

Time waveform and spectrum of some AE samples on different nodes are shown in Figure 3. The time waveform was stochastic and the amplitude did not change regularly with the grinding time elapse. In the frequency domain, the majority of the energy was concentrated in a characteristic frequency band centered by 180 KHz for all nodes. However, the energy distribution pattern was quite different from each other. As analyzed in Section 2, AE signal of grinding process is a superposition of a series of b-AE. Irregular abrasive grains rub, plough or cut the workpiece surface simultaneously in the grinding process. The waveform of different kinds of b-AE overlaps in time domain. Therefore, damping oscillation pattern of a specific b-AE cannot be identified and AE signals are stochastic in time domain. However, different kinds of b-AE possess a different characteristic frequency band. Predominant crack modes of the ground brittle material may be various with the deterioration of wheel condition. It results in the changes of superposition relationship of b-AE components with different mechanisms. Therefore, AE spectrums were various for different grinding time phases as shown in Figure 3. For AE samples belonging to a specific node, spectrums were quite similar. It manifested that statistical characteristics of AE signals were stable for a specific state of wheel condition.

Linear discriminant analysis (LDA) [37] was used to further reveal the variety of AE spectrums with grinding time elapse. LDA is a supervised dimensionality reduction method. The basic idea is to project all samples on a low-dimensional feature space on the premise that samples in the same class are as close as possible and at the same time different classes are far away from each other as much as possible. A linearly separable dataset including N classes can be totally separated in a N−1 dimensional feature space.

**Figure 4:**
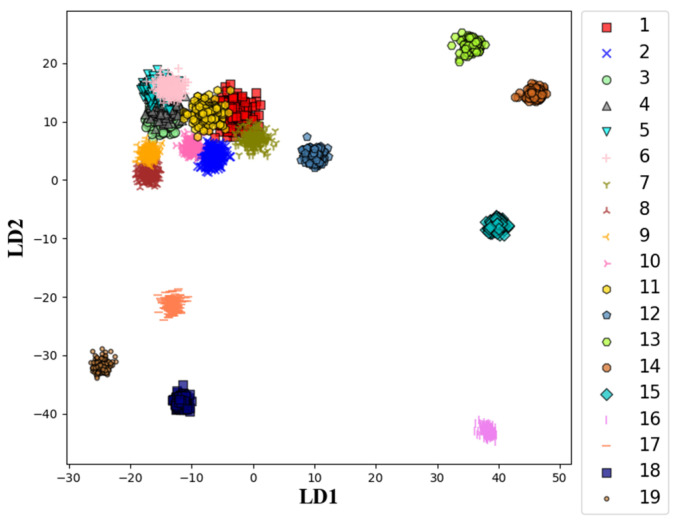
The projection of AE spectrums on a two-dimensional feature space.

The generation mechanism determines the superposition and linear separability of AE signals of grinding brittle material. Therefore, LDA can be used to evaluate the variety of AE spectrums for all the observation nodes. Taking the order number of observation nodes as labels, spectrums of all AE samples were projected on a two-dimensional feature space by LDA as shown in Figure 4. AE samples belonging to a certain node are drawn with the same color and the same mark in the figure. Color and mark used for the nineteen nodes are listed in the figure legends. AE samples were chronologically acquired on the nineteen nodes during experiments and they were assigned into nineteen classes corresponding to various states of wheel condition. Samples from the same node are concentrated in a small local area because of their quite similar spectrum pattern. The distances between various classes manifested their differences. Classes corresponding similar wheel states are close or overlapped in the two-dimensional feature space. As shown in Figure 4, sample sets before the 12th node were tangled with each other in the projection. It manifested that the wheel state was relative stable before the time point of the 12th node. The subsequent nodes were all far away from the stable range, and moreover, there were distinct distances between them. Wheel state dramatically changed with the time elapse. The results coincide with the evolution of wheel topography discussed in the above section.

## 4. Wheel State Classification Based on Original AE Time Waveform

### 4.1. CNN Model of Wheel State Classification

CNN is a kind of feedforward neural networks which contains convolution computation and has deep structure. It is one of the representative algorithms of deep learning. CNN is originally applied in image classification [38]. The existing literatures presented the successful application of CNN in residual life prediction and tool condition monitoring [39,40]. In order to directly transplant the model structure used in image tasks, the majority of them took time-frequency images or other two-dimensional data as the model input. CNN has the ability of representation learning and can shift invariant classification of input information according to its hierarchical structure, so it is also known as shift invariant artificial neural networks [41]. The advantage of CNN is coincided with the convolution form of AE signals during grinding brittle materials as mentioned in Section 2. Therefore, one-dimensional AE time waveform is taken as the input of CNN model in the paper. CNN integrates feature extraction and classification, and its deep network structure endows CNN with the capability of deconvolution of AE features mixed in time domain.

### 4.2. Multi-Class Classification of Wheel State Based on CNN

The original time-domain data was used as the input data and the node number was used as the label for training. Therefore, there were 19 sets of samples, and each set of samples has 2000 pieces of data. In total, 30% of the data is randomly selected as a test set, and the rest is the train set.

**Figure 5:**
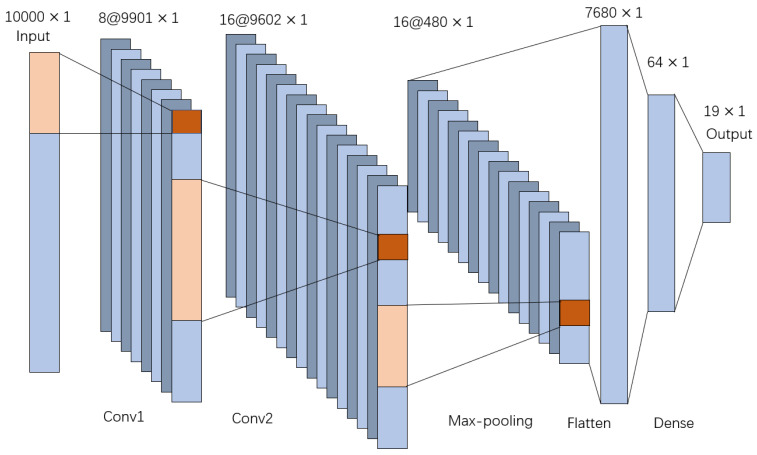
The process of the convolutional neural network (CNN).

The process of the CNN used for wheel state classification is shown in Figure 5. Comprehensively considering the accuracy of the model and the time required for training, a network with two convolutional layers and one pooling layer was selected. According to the time domain characteristics of the signal, in order to make each convolution kernel capture local features as much as possible but not ignore the relationship between time series, the convolution kernels of 100 and 300 lengths are selected by trying combinations of different parameters. The first convolutional layer has 8 kernels of length 100, and the second convolutional layer has 16 kernels of length 300, followed by the pooling layer, and the maximum pooling layer with the size of 20 is chosen. In this layer, the size of the output of the second convolutional layer is reduced to 1/20 of its original size. Then, the data are flattened, and, finally, there are two full connection layers that connect the output layer.

**Figure 6:**
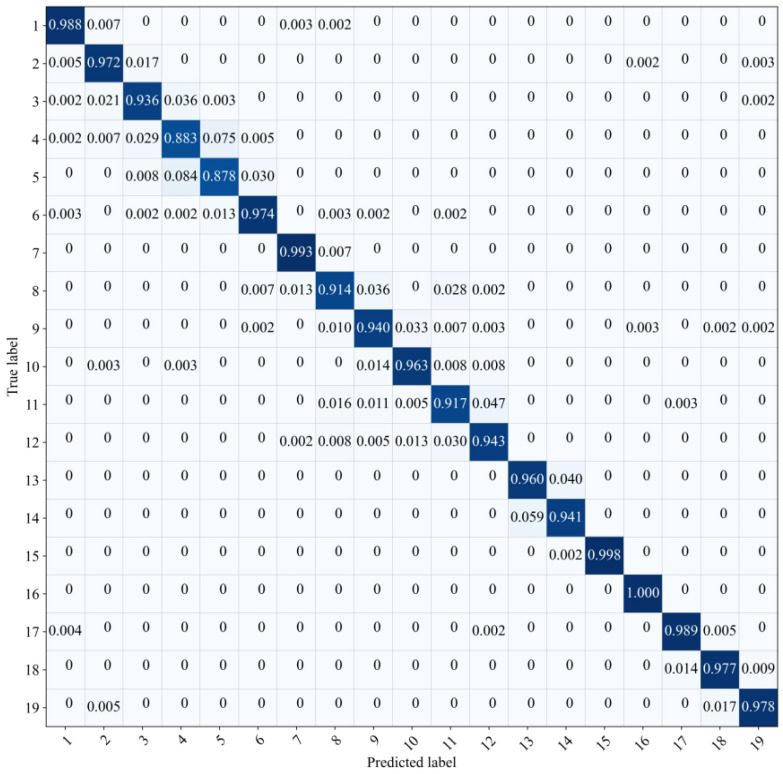
The confusion matrix of the results.

The accuracy of the trained model is represented by the confusion matrix, it is mainly used to compare the predicted label with the true label. Each column of the confusion matrix represents the predicted label; each row represents the true label to which the data belongs. As shown in Figure 6, except for group four and five, the prediction accuracy is over 0.90. Taking original AE time waveform as the input, CNN performed well on multi-class classification of wheel state. For further understanding the performance of the CNN model, the following will focus on visualization technique that gives insight into the function of intermediate layers.

### 4.3. Visualization and Analysis of Convolution Output

**Figure 7:**
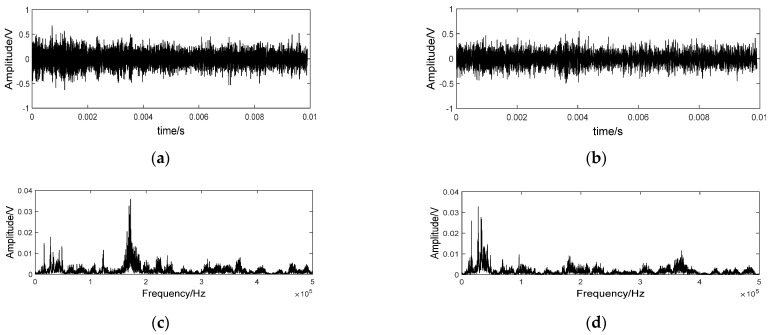
The output of different convolution kernel of the 5th node. (**a**) the output of the 1st kernel; (**b**) the output of the 2nd kernel; (**c**) the spectrum of the waveform in (**a**); (**d**) the spectrum of the waveform in (**b**).

**Figure 8:**
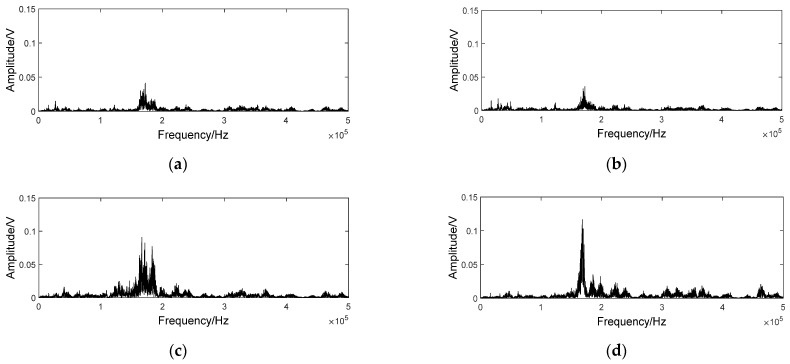
The output of different nodes through the same kernel. (**a**) the 3rd node; (**b**) the 5th node; (**c**) the 13th node; (**d**) the 17th node.

The visualization of intermediate layers clarified the performance of CNN. An AE sample of the 5th node was put into the trained model, and the outputs of the first two kernels of the first convolutional layer are shown in Figure 7a,b, respectively. They were random as the original input. It is hard to recognize the difference on time domain. Therefore, Fourier transform was used for further comparison. Spectrums of the two output signals are also shown in Figure 7c,d, respectively. The obvious difference in energy distribution manifests that the two kernels are focused on signal characteristics of different frequency domain. That is to say, characteristics of different AE components originally mixed in time domain were extracted and separated by diverse kernels. Therefore, more distinct changes can be recognized without the disturbance of other components. It is verified by comparing the outputs of some samples from different nodes as shown in Figure 8. With the deterioration of wheel condition, characteristics in frequency span 150–250 kHz were dramatically changed.

## 5. LSTM for Wheel State Classification

For common classification problems, the order of training samples is inessential for model training and classification accuracy. However, for tool condition classification and regression, tool condition undoubtedly deteriorates with the time elapse. Wheel’s previous state will impact its subsequent performance. Correspondingly, characteristics of samples evolve gradually and there exists some kind of relationship between forward and backward samples.

Considering the time-varying correlation of AE samples, long short-term memory (LSTM) [42] network is quite suitable for wheel condition monitoring. LSTM is a special kind of recurrent neural network (RNN) [43,44], and it has capable of learning long-time dependencies. LSTM replaces the nodes in the hidden layers of RNN with a special memory cell. By using this memory cell which selectively retains or discards the past time step information, LSTM can model the long time-dependent sequence and is more suitable for the modeling of grinding wheel wear process.

### 5.1. Basic Principle of LSTM

**Figure 9:**
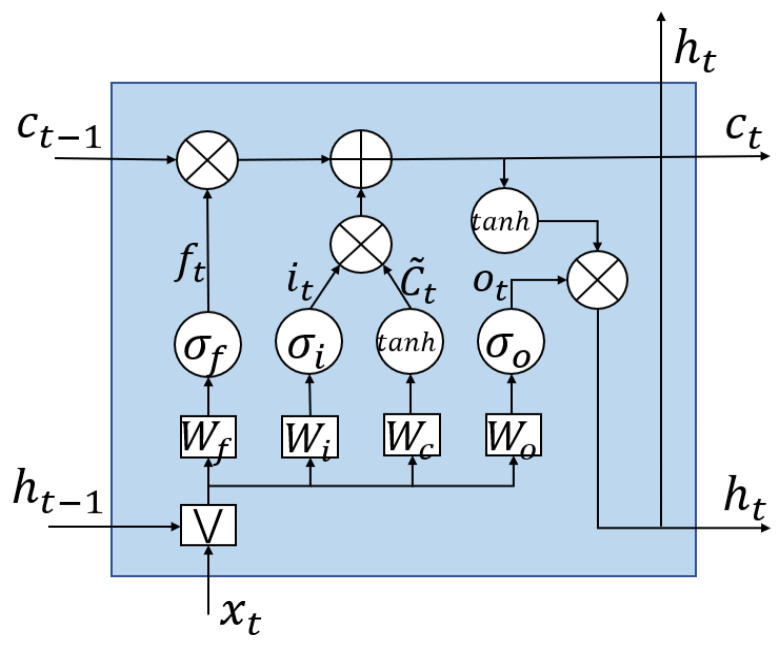
Basic structure of long short-term memory (LSTM) cell.

The special composite memory cell is shown in Figure 9. This special node has two state transmission connections between adjacent time steps. It calculates the output $h_{t}$ and the internal state $c_{t}$ of the current time step according to the state $c_{t-1}$ and $h_{t-1}$ transmitted from the previous time step and the input $x_{t}$, then passed them to the next time step. There are three Sigmoid activation function gates in LSTM cell, which are forgetting gate $\sigma_{f}$, input gate $\sigma_{i}$ and output gate $\sigma_{o}$. The state $c_{t-1}$ transmitted from the previous time step and the input of at the current time step consists of column vector ${\left[\begin{array}{cc} x_{t} & h_{t-1} \end{array}\right]}^{T}$, then this vector is multiplied by the weight matrix $W_{f}$. This process determines how much the information $c_{t-1}$ of the previous time step should be retained. With forget gates, the equation to calculate output $f_{t}$ is shown in Equation (4), where is $b_{f}$ is the bias parameter [42].

(4) $$f_{t}=\sigma \left(W_{f}\left[\begin{array}{c} x_{t}\\ h_{t-1} \end{array}\right]+b_{f}\right)\;\;$$

The calculation of state C in the current time step is shown in Equations (5)–(7), where $W_{i}$ is the input weight matrix, $W_{c}$ is the state weight matrix, $i_{t}$ is the output of the input gate. Here ${\tilde{c}}_{t}$ is the output of the tanh function gate, the vectors $b_{i}$ and $b_{c}$ are bias parameters.

(5) $$c_{t}=f_{t}*c_{t-1}+i_{t}*{\tilde{c}}_{t}$$

(6) $$i_{t}=\sigma \left(W_{i}\left[\begin{array}{c} x_{t}\\ h_{t-1} \end{array}\right]+b_{i}\right)\;\;\;\;\;$$

(7) $${\tilde{c}}_{t}=\tan h\left(W_{c}\left[\begin{array}{c} x_{t}\\ h_{t-1} \end{array}\right]+b_{c}\right)$$

The output of current time step are calculated as shown in Equations (8) and (9), where $W_{o}$ is the output weight matrix, $o_{t}$ is the output of the output gate.

(8) $$h_{t}=o_{t}\cdot \tan h\left(c_{t}\right)$$

(9) $$o_{t}=\sigma \left(W_{o}\left[\begin{array}{c} x_{t}\\ h_{t-1} \end{array}\right]+b_{o}\right)$$

### 5.2. Regression Analysis of Wheel State Based on LSTM

The AE signal acquired on each node was divided into data frames with 10,000 sampling points, which were converted to the corresponding spectrums by Fourier transform, consisting of the data set. The sequence order of all the 19 nodes were taken as data labels. The sample data set was divided into training set (75%) and test set (25%). The deterioration of wheel condition is a continuous process. Additionally, it is appropriate to be modeled as a regression instead of classification. The LSTM network was trained with the labelled samples of the training set. Considering continuous evolution of wheel condition, regression instead of classification was tried in the paper. The loss function was the mean square error (MSE), the optimizer was SGD and the learning rate was set to 0.001. The selection of hyperparameters such as network structure parameters and time steps will affect the recognition ability of the network. 25% samples of training set was randomly selected as validation set. Different hyperparameters (hidden layers from 2 to 5 and nodes of hidden lays from 20 to 80) were adopted to train the LSTM network and the best one was fixed according to the model prediction error on the validation set. The final selected model with the smallest error consisted of four layers: the input layer, two LSTM hidden layers, and one output layer. Additionally, the hidden layer consisted of 40 LSTM units.

**Figure 10:**
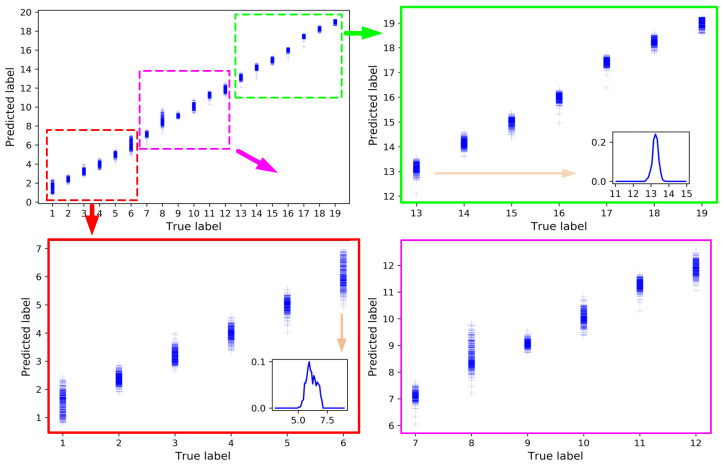
Prediction results LSTM model.

**Figure 11:**
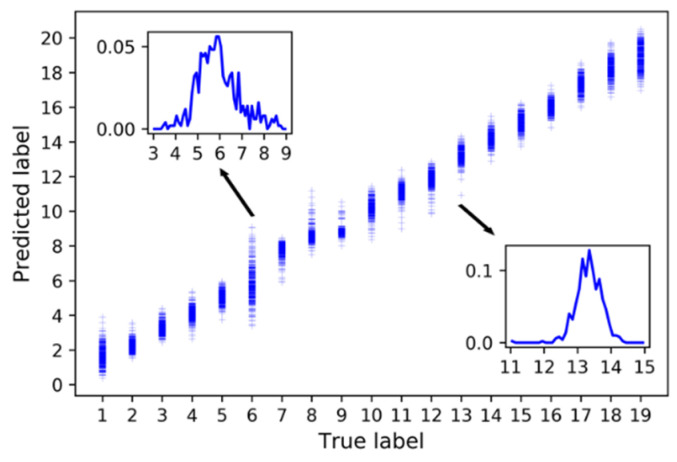
Prediction results BPNN model.

**Figure 12:**
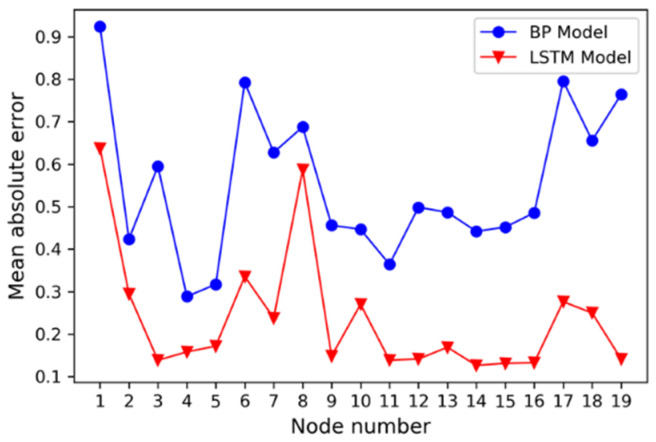
Model error comparison.

After 15 steps of training, the mean absolute error (MAE) of the training set converged to 0.0322, and the MSE converged to 0.1328. The MAE and MSE of the test set were 0.1055 and 0.2361, respectively. Additionally, the results on the test set are shown in Figure 10. The upper left picture is the integrated results for all the nineteen nodes. It was divided into three parts drawn in the other three pictures just for the sake of clarity. The predicted label was continuous value for regression. Except the 1st and the 8th nodes, the predicted labels of samples on the other nodes were concentrated on the true label value. For further comparison, the probability density function of predicted label of the 6th and 13th nodes are also displayed on the blank of the pictures. As shown in Figure 10, the accuracy of the models is relatively low at the 6th node and relatively high at the 13th node. The result of the corresponding optimal traditional BPNN network in the test set after 1000 steps of training are shown in Figure 11, and the corresponding MAE and MSE were 0.5345 and 0.4807, respectively. The probability density function of predicted label by the BPNN network of the 6th and 13th nodes are also displayed in Figure 11. The MSE of the LSTM network model was reduced by 78.1% and the MAE was reduced by 50.9% compared with the BPNN model. The sub-node test errors of the two models are shown in Figure 12. The performance of LSTM was better than BPNN on all nodes. Additionally, there were low MAE except the 1st node and the 8th node.

## 6. Conclusions

AE-based monitoring is a promising technique for tool condition monitoring. This paper is dedicated to AE-based condition monitoring of diamond wheel during grinding brittle materials. Comparing with conventional grinding process, a consistent grinding process is required to avoid unpredicted catastrophic damages on or under the ground surface of brittle material. AE Feature representation is the key to realize high monitoring accuracy. In order to select appropriate features, various signal processing methods and feature selection theories have been tried and studies. However, nowadays, the trial-and-error method is still the mainstream for AE feature representation.

In this paper, a convolution form of AE signals under brittle grinding regime was proposed for deep understanding characteristics of AE signals during grinding brittle materials. The convolution form of AE signals in time domain exactly corresponds with the principle of CNN. Therefore, original time waveform of AE can be directly put into the deep network for wheel state classification. Visualization of intermediate output of CNN model manifested that different convolutional kernels put emphasis on different AE mechanisms. Experimental results verified that detailed state division in a wheel’s whole life cycle can be realized.

Different from the overlapping in time domain, AE components with different mechanisms were theoretically proved to be separated in frequency domain. Predominant AE mechanisms will change with the deterioration of wheel state. Therefore, AE spectrum pattern and the energy distribution will change correspondingly. It is easy to catch the evolution from frequency domain. Considering the time sequence of AE signals during grinding, a regression prediction model based on long short-term memory (LSTM) was built with AE spectrums as the model input and satisfactory prediction accuracy was acquired.

Taking original time waveform and frequency spectrum as model input, respectively, CNN and LSTM performed well on multi-class classification and regression. Deep network structures make it possible of learning feature representation automatically. Avoiding the blindness and arbitrary of artificial features, decision-making and evaluation will be more objective and accurate. The future work will focus on the prediction of residual useful life of grinding wheel. It will be helpful for practical quality control and efficiency improvement of grinding process to be conducted.

## Figures and Tables

**Table 1:** Parameters of the cup wheel, workpiece and the grinding process.

Cup wheel	abrasive	bond	Diameter	mesh grain size	Concentration			
	diamond	Resin bond	50 mm	400#	100%			
workpiece	Young’s modulus	Shear modulus	Modulus of rupture	Knoop hardness (100 g load)	Density	Softening point	Specific heat	Thermal conductivity
	73 GPa	31 GPa	52.4 MPa	522 kg/mm^2^	2.2 g/cm^3^	1585 ℃	0.770 J/(g K)	1.38 W/(m K)
Grinding processing	observation nodes	volume of material removal (${\mathrm{cm}}^{3}$)	spindle speed (n/s)	cutting depth (µm)	grinding grating interval (mm)	workpiece speed (mm/min)		
	1~19	0.1:0.1:1.9	50	5	15	600		

## Nomenclature

AE	acoustic emission
b-AE	burst-type AE
c-AE	continuous-type AE
CNN	convolution neural network
LSTM	long short-term memory
x(t)	AE signal
ω	frequency factor
$X_{half}\left(\omega \right)$	Fourier transform of x(t) (the positive-frequency part)
e	the base of natural logarithms
δ	the delta function
u(t)	the unit step signal
α	the damping factor
A	the amplitude of b-AE
$h_{t}$	the output of LSTM cells
$c_{t}$	the internal state of LSTM cells
$x_{t}$	the input of LSTM cells
$f_{t}$	the output of the Sigmoid gate of forgetting
$i_{t}$	the output of the Sigmoid gate of input
$o_{t}$	the output of the Sigmoid gate of output
${\tilde{c}}_{t}$	the output of the tanh gate
$b_{f}$	the bias parameter of the Sigmoid gate of forgetting
$b_{i}$	the bias parameter of the Sigmoid gate of input
$b_{c}$	the bias parameter of the tanh gate of the LSTM cell
$b_{o}$	the bias parameter of the Sigmoid gate of output
$W_{f}$	the forgetting weight matrix
$W_{i}$	the input weight matrix
$W_{o}$	the output weight matrix
$W_{c}$	the state weight matrix of the LSTM cell
σ	the Sigmoid function
$\sigma_{f}$	the Sigmoid gate of forgetting
$\sigma_{i}$	the Sigmoid gate of input
$\sigma_{o}$	the Sigmoid gate of output
